# A cell-loss-free concave microwell array based size-controlled multi-cellular tumoroid generation for anti-cancer drug screening

**DOI:** 10.1371/journal.pone.0219834

**Published:** 2019-07-25

**Authors:** Sang Woo Lee, Soo Yeon Jeong, Tae Hoon Shin, Junhong Min, Donghyun Lee, Gi Seok Jeong

**Affiliations:** 1 Biomedical Engineering Research Center, Asan Institute for Life Sciences, Asan Medical Center, Seoul, Korea; 2 School of Integrative Engineering, Department of Biomedical Engineering, Chung-Ang University, Seoul, Korea; University of South Florida, UNITED STATES

## Abstract

The 3D multi-cellular tumoroid (MCT) model is an *in vivo*-like, avascular tumor model that has received much attention as a refined screening platform for drug therapies. Several types of research have been efforted to improve the physiological characteristics of the tumor microenvironment (TME) of the *in vivo*-like MCTs. Size-controlled MCTs have received much attention for obtaining highly reproducible results in drug screening assays and achieving a homogeneous and meaningful level of biological activities. Here, we describe an effective method for fabricating the size-controlled *in vivo*-like MCTs using a cell-loss-free (CLF) microwell arrays. The CLF microwell arrays was fabricated by using the simple operation of laser carving of a poly (methyl methacrylate) (PMMA) master mold. We also demonstrated the biophysicochemical effect of tumor microenvironment (TME) resident fibroblasts through the expression of TGFβ, αSMA, Type I-, IV collagen, angiogenesis related markers on tumorigenesis, and confirmed the drug response of MCTs with anti-cancer agents. This technology for the fabrication of CLF microwell arrays could be used as an effective method to produce an *in vitro* tumor model for cancer research and drug discovery.

## Introduction

Recently, multi-cellular tumoroids (MCTs) have received much attention for the study of a refined screening platform for drug therapies [1, 2]. The physiological characteristics of the three-dimensional (3D) MCTs closely resemble avascular tumor nodules, micro-metastases, and inter-vascular regions of large solid tumors [3–5]. Conventional two-dimensional (2D) platforms are well established and easy to use for these applications [6]. However, the absence of 3D cell-cell and cell-matrix interactions can obscure experimental observations and result in misleading and contradictory results during drug screening [7]. Indeed, the lack of the complex 3D extracellular matrix (ECM) network in monolayer culture can affect drug testing results [7, 8].

To develop *in vivo*-like MCTs, various research efforts have been made to improve the physiological characteristics of the tumor microenvironment (TME) [3–5, 9, 10]. These include aggregating cells or growing cells in stirred culture systems, such as spinner flasks [3], spontaneous aggregation in non-adhesive culture plates [11, 12] and stirred-tank bioreactors [13]. However, these techniques usually display a broad size distribution and are not suitable for the formation of a controlled TME [14]. Indeed, size-controlled MCTs have received much attention for obtaining highly reproducible results in drug screening assays and achieving a homogeneous and meaningful level of biological activities [15].

Several studies have utilized different techniques to generate the size-controlled MCTs with the *in vivo*-like complex architecture [5, 9, 10, 16–18]. Hanging drop cultures [9] and microfabricated microstructures [19, 20] are often used to generate size-controlled MCTs. Among them, 3D tumor formation using microwell arrays is an emerging technology for the stable, scalable and reproducible production of MCTs with the real-time observation of tumor progression [21–23]. Furthermore, microfluidic devices are suitable tools for drug screening systems with *in vivo*-like TMEs [24]. However, improvement is needed for this process, such as simplifying the washout and reducing the loss of applied cells, for the advanced use of this platform.

Many researchers proposed several approaches to minimize cell loss in the developed microwell arrays and to allow the efficient formation of 3D culture products until recently [1, 13, 21–35]. In this study, we describe a 3D MCTs formation method using novel concave microwell arrays with a cell-loss-free (CLF) platform. For the novel concave microwell array with CLF platform, we designed the microwell array to improve the unbalanced cell-cell interaction on the initial aggregation process of the cells and the low plate structure, and to minimize the space between microwells to prevent false trapping of cells. Then, we made PDMS mold to fabricate the formation of 3D MCTs using a modified acrylic plate by a carved pattern created using a laser cutter. Using the CLF microwell array, more than 600 spheroids of size-controlled MCTs can be spontaneously fabricated simultaneously. During the MCTs formation, ECM (type I and IV collagen) and angiogenesis-related markers were shown higher expression in the tri-culture with fibroblasts than in co-culture without fibroblasts. In addition, drug screening of the 3D MCTs using Taxol and Gemcitabine, which have been used clinically as anti-cancer agents for non-small cell lung cancer, was performed. Using this advanced CLF concave microwell array, MCTs could improve the understanding of tumorigenesis and the development of novel therapeutics.

## Materials and methods

### Fabrication of the CLF concave microwell array

**Fig 1:**
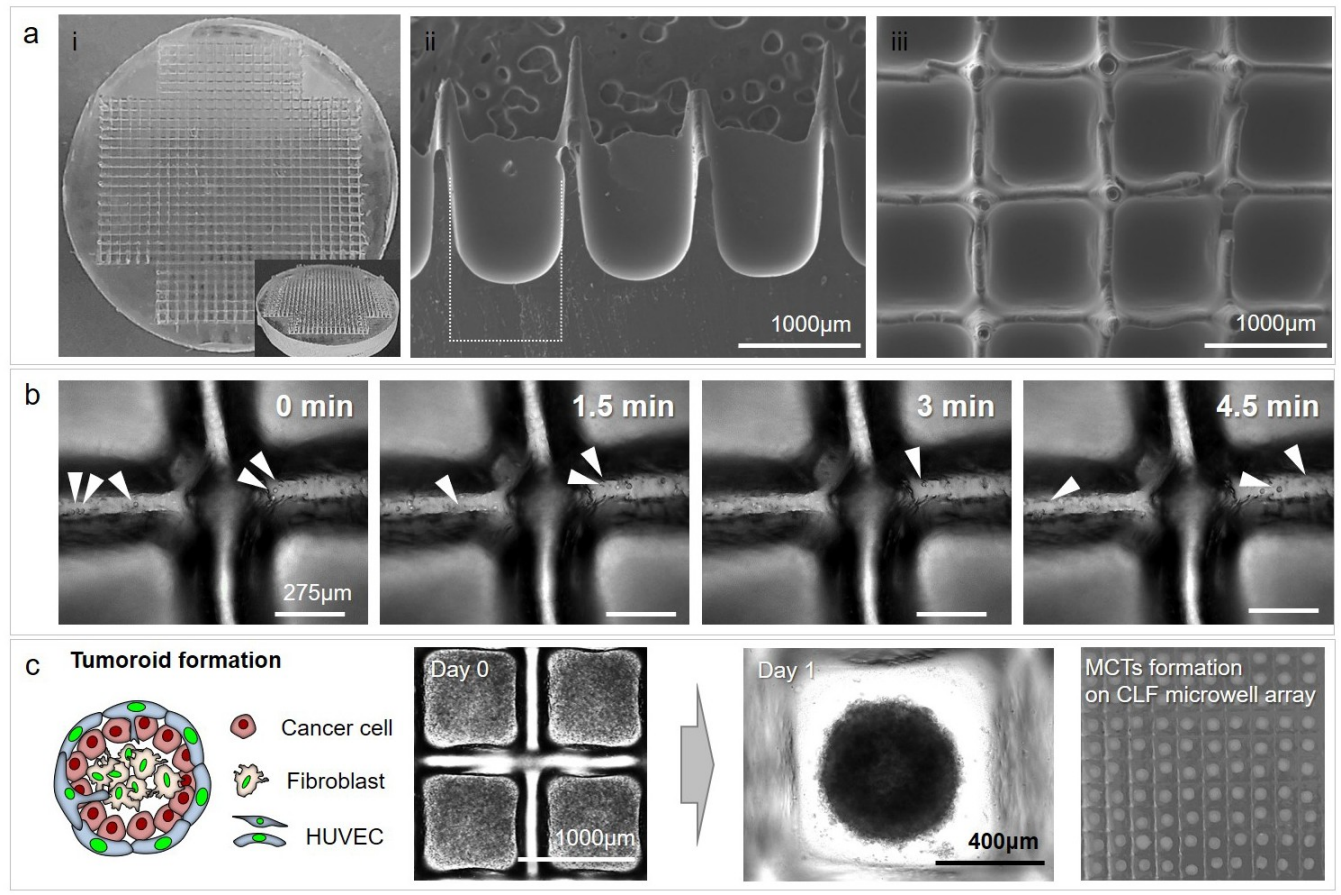
Fabrication of cell-loss-free (CLF) concave microwell array for multi-cellular tumoroids (MCTs) formation. (a) The PDMS CLF concave microwell array was fabricated into a size suitable for the cell culture plate (i). The meniscus concave shape bottom within the microwells was confirmed by SEM image (ii). It was confirmed that the PDMS concave microwell array consisting of a microwell with a wall 1 mm high for separating each well (iii). (b) Cell sliding along spiky walls by gravity after initial cell trapping in CLF microwell array. (c) Compact spheroids by the cell mixture with cancer and fibroblasts, and obtainment of a large number of MCTs in the fabricated CLF concave microwell array.

To fabricate the mast mold, a poly(methyl methacrylate) (PMMA) sheet (5 mm of thickness) was carved with a rectangular pattern approximately 1.5 mm in depth using a laser carving machine (BCL-1006X, bodor, Jinan city, China) (S1 Fig). The poly(dimethylsiloxane) (PDMS; Sylgard 184, Dow Chemical Co., MI, USA) microstructure was replicated from a PMMA master mold for fabricating the PDMS well array. After peeling off the PDMS well array, the mixture of a PDMS prepolymer, containing a PDMS precursor and curing agent in a 10:1 ratio, was poured into the PDMS well array. Then, approximately 80% of the mixture of PDMS prepolymer was removed. The surface tension of the PDMS prepolymer remaining in the PDMS well array caused a self-organized meniscus to be formed in the cylindrical microwells (S1 Fig and Fig 1(a(ii)); white dotted line indicates cylindrical microwells of the PDMS well array); the contact angle between the prepolymer meniscus and the PDMS sidewall was determined to be approximately 20° [26, 29, 36]. This fabrication procedure produced a large number of the CLF concave wells simultaneously (Fig 1(a(i) and 1(a(iii))) without using any specialized tools or complicated procedures. Fig 1(a(ii)) shows scanning electron microscopy (SEM) images, demonstrating the hemispherical structure of the microwell.

### Cell culture and generation of multi-cellular tumoroids

For the cell culture, the A549 cell line (a human lung adenocarcinoma cell line, ATCC; CCL-185, Manassas, VA, USA) was cultured in RPMI-1640 (Thermo Fisher Scientific, MA, USA). The MRC-5 cell line (a human lung fibroblast cell line, ATCC; CRL-171, Manassas, VA, USA) was cultured in Dulbecco’s modified Eagle’s medium (Thermo Fisher Scientific) with 10% fetal bovine serum (FBS, Biowest, MO, USA) and 1% antibiotics (Thermo Fisher Scientific). HUVECs (human umbilical vein endothelial cells, ATCC; CRL-1730, Manassas, VA, USA) were grown in ECM (ScienCell, California, USA) as the endothelial cell medium, consisting of 500 ml of basal medium, 25 ml of fetal bovine serum (FBS, Cat. No. 0025), 5 ml of endothelial cell growth supplement (ECGS, Cat. No. 1052) and 5 ml of penicillin/streptomycin solution (P/S, Cat. No. 0503). To generate the multi-cellular tumoroids, the CLF concave microwell array was coated with 4% bovine serum albumin (BSA) (Thermo Fisher Scientific, MA, USA) using a micropipette for 24 hours. The A549 (5000 cells/well in microwell array), MRC-5 cells (5000 cells/ well in the microwell array) and HUVECs (2500 cells/ well in the microwell array) were prepared as suspensions in ECM medium. After removing the BSA solution from the concave microwell array through aspiration, the CLF concave microwell array was rinsed with phosphate-buffered saline (PBS, Thermo Fisher scientific) solution, and A549 + HUVECs mixture or A549 + MRC-5 + HUVECs mixture were gently plated on the concave microwell array. It was centrifuged at 1,000 revolutions per minute (RPM) for 1 min using a centrifuge to settle the mixtures into the bottom of the microwells. After incubation for 3 h at 37°C, the CLF concave microwell array was gradually supplied with new nutrition by exchanging ECM medium twice a day. The interactions among the cancer cells, fibroblasts, and HUVECs were monitored daily using an optical microscope (EVOS; Life Technologies, Carlsbad, CA, USA).

### Fluorescence tracker labeling

MRC-5 cells were fluorescently labeled using PKH26 (red; Sigma-Aldrich). The cells were placed in a conical tube at a cell density of 2 × 10^6^ cells and washed once using medium without serum. After centrifugation (400 × *g*) for 5 min, the supernatant was carefully aspirated to leave 25 μl of supernatant. The cells were suspended by adding 150 μl of Diluent C solution to the pellet. Immediately, the dye solution was prepared with Diluent C, which was added with a PKH ethanolic dye solution (2 μl). The cells with the Diluent C solution and the prepared dye solution were rapidly mixed and gently suspended. After incubation at room temperature for 5 min, an equal volume of a suitable protein solution (1% bovine serum albumin) was added to stop the staining, and the resulting solution was incubated for 1 min to allow binding of the excess dye. The solution with the stained cells was centrifuged at 400 × *g* for 10 min at 20–25°C, and the supernatant was removed. The cell pellet was re-suspended in 10 ml of complete medium and transferred to a fresh sterile conical tube. Cells were centrifuged at 400 × *g* for 5 min at 20–25°C and washed twice with 100 ml of complete medium to ensure the removal of any unbound dye. After the final wash, the fluorescent dye-stained cells were used in experiments to confirm their position and migration.

### Immunofluorescence assay

The *in vitro* MCTs that formed in the CLF concave microwell array were fixed with 4% paraformaldehyde (Sigma-Aldrich) for 10 min at room temperature; 100% methanol (chilled at −20°C) was added, followed by incubation for 5 min at room temperature. The CLF concave microwell array was then washed five times with ice-cold PBS (Thermo Fisher scientific). For permeabilization, MCTs were incubated using PBS containing 0.1% Triton X-100 (Sigma-Aldrich) for 40 min at room temperature and blocked with 2% bovine serum albumin (BSA, Sigma-Aldrich) in 0.1% Tween 20 (Sigma-Aldrich) and PBS (PBST) for 45 min. The MCTs were incubated with primary antibody in 1% BSA in PBST overnight at 4°C. The spheroids were washed with PBS for 5 min and incubated with secondary antibodies (Alexa 488, Alexa 647 (Abcam, Cambridge, UK)) for 4 h at room temperature. The nuclei were counterstained with 4’,6-diamidino-2-phenylindole dihydrochloride (DAPI, Invitrogen, Carlsbad, California, USA). Primary antibodies against transforming growth factor-beta (TGFβ), α-smooth muscle actin (SMA), collagen-I and –IV, matrix metalloproteinase (MMP)-1 and -9, VE-cadherin, CD31, von Willebrand factor (vWF) and vascular endothelial growth factor (VEGF) were purchased from Abcam. Immunostained MCTs were isolated onto confocal dishes to capture the fluorescent images from the CLF concave microwell array. Fluorescence images were obtained using a fluorescence microscope (EVOS) and a confocal microscope (Zeiss LSM780, Carl Zeiss AG, Oberkochen, Germany). The confocal images were analyzed using ZEN Microscope Software (Carl Zeiss AG, Oberkochen, Germany).

### Observation of MCTs using scanning electron microscopy (SEM)

*In vitro* MCTs in the CLF concave microwell array were fixed with 2.5% glutaraldehyde (Sigma-Aldrich) in deionized water (DW) for 1 h at room temperature and washed five times with DW. Secondary fixation was performed using 1% osmium tetroxide (Sigma-Aldrich) in DW for 1 h. The fixed MCTs were dehydrated using a graded series of ethanol (25%, 50%, 75%, 95%, and 100%). After dehydration, the MCTs were washed three times with *tert*-butyl alcohol (Sigma-Aldrich) and frozen at −70°C. The frozen MCTs were lyophilized until the *tert*-butyl alcohol had evaporated and were mounted on a specimen stub, coated with palladium alloy, and observed under a scanning electron microscope (AIS 1800C, SERON Tech, Korea).

### Assessment of the anti-cancer drug response

MCTs that formed similarly to the *in vivo* tumor tissue by MRC-5 cells were used to assess the response to anti-cancer drugs. Cell mortality in the CLF concave microwell array was assessed by administering paclitaxel (3 μM) (Taxol, Sigma-Aldrich, USA) and gemcitabine (3 μM) (Sigma-Aldrich, USA) as anti-cancer drugs for NSCLC for 4 days. For this purpose, each single agent, a combination of paclitaxel + gemcitabine, and a mock group for comparison were diluted in the 3D culture medium ECM (ScienCell, California, USA) after dilution with dimethyl sulfoxide (Sigma-Aldrich). The anti-cancer drugs were administered into the concave microwell array containing MCTs with a high circularity score (≥ 0.7). Analysis of cellular mortality to evaluate the drugs was performed by fluorescence staining using the live/dead kit described previously. Morphologies and fluorescence images of MCTs were observed using a fluorescence microscope (EVOS; Life Technologies, USA) on days 2 and 4 after initial administration of drugs.

### Assessment of cell viability for the evaluation of drugs

After 2 days of MCTs formation, two were re-plated into each well of a 96-well plate and incubated in a humidified incubator at 37°C and 5% CO2. The anti-cancer drugs (Taxol (paclitaxel), Gemcitabine and a combination of Taxol + Gemcitabine) were administered to the 96-well plate containing MCTs for 4 days. Then, the cell counting kit-8 (CCK-8; Dojindo Molecular Technologies Inc., Rockville, MD, USA) solution was added to each well, and the plate was incubated for 5 h in the incubator. Cell viability was measured at 450 nm using a microplate reader (Sunrise, TECAN, Männedorf, Switzerland). Cell viability was performed on days 2 and 4 after initiation of drug administration. Each drug administration group was analyzed four times.

Additionally, to identify the drug response, apoptotic cell death was analyzed using the Annexin V–FITC apoptosis detection kit (cat no. APOAF, Sigma–Aldrich) on the fourth day after the onset of the administration of the anticancer drugs. Stained images were obtained with a confocal scanning microscope LSM780 (ZEISS, Germany).

### Statistical analysis

The circularity, diameter and fluorescence intensity of the MCTs were determined by ImageJ Software (1.46 ver, NIH, USA). Quantitative data are presented as the mean ± standard deviation. Group differences were assessed by paired t-tests or one-way and two-way ANOVAs using ORIGIN 2018b (OriginLab Corp., MA, USA). Statistical significance was set at *p* > 0.05(ns), *p* < 0.05(*) and *p* < 0.005(**).

## Results

### Fabrication of the CLF concave microwell array using a laser cutter for MCTs formation

A poly(methyl methacrylate) (PMMA) sheet was carved with a square pattern using a laser carving machine (S1(i) Fig). Throughout the process, the PMMA master mold was fabricated without any other processes. After PDMS base mold replication (S1(ii) Fig), prepolymer PDMS was added to the PDMS base mold for fabricating the CLF concave microwell array (S1(iii)–S1(vii) Fig). Although the partition wall between the concave microwells is approximately 100 μm wide, the shape of the partition wall has a spiky edge (Fig 1(a(ii)) and S2(a) Fig, white arrowheads) which allows all cells in the suspension to be trapped in the concave microwells by gravity (Fig 1(c, day 0), S2(a) and S2(b) Fig). The CLF concave microwell array (diameter: 1 mm) was confirmed by SEM image (Fig 1(a(ii) and 1(a(iii))). The size and structural design can be adjusted through technology established in this study. It has a great advantage and was characterized by SEM images (S3 Fig). Fig 1(a(iii)) shows the top view of the CLF concave microwell array. A549 and MRC-5 cells were labeled with a fluorescence tracker PKH67(green) and PKH26(red), respectively. To evaluate the minimization of cell loss by the spiky wall in the CLF microwell array, cells were trapped and observed for 30 min. The cells (white arrowheads on Fig 1(b) and S4(a) Fig) identified in the captured images for 5.5 min were trapped in each well after initial cell trapping. And, in the images captured at 3 focus positions (top, middle and bottom position) for 24 hours after trapping the tracker labeled cells using a fluorescence microscope, whole trapping cells stably embedded and aggregated in the bottom layer of microwells (S4(b) Fig). The reduction of cell area of similar size was also identified by cell aggregation (S4(c) Fig). Trapping cells flowed naturally along the spiky walls by gravity and were stably embedded into microwells by minimizing cell loss (S1 Movie). A549 human lung carcinoma cells, HUVECs human vascular endothelial cells, and MRC-5 human lung fibroblasts which served as cancer resident fibroblasts, were pre-mixed at a ratio of 2:1:2 and plated in the CLF concave microwell array to form MCTs (Fig 1(c), day 0). The cell mixture was settled and isolated in each well. By the influence of the CLF architecture, the aspiration step for removing residual cells was omitted, and the most cells were used for MCTs formation. The cell mixture formed compact spheroids (diameter: 500 ~ 550 μm) after 1 day, and a large number of MCTs were obtained using the fabricated PDMS mold (Fig 1(c), day 1). As a result, it was confirmed that the CLF concave microwell array is useful for forming a large number of MCTs (Fig 1(c)).

### Morphological analysis of MCTs formed by cancer cells and fibroblasts in the CLF concave microwell array

**Fig 2:**
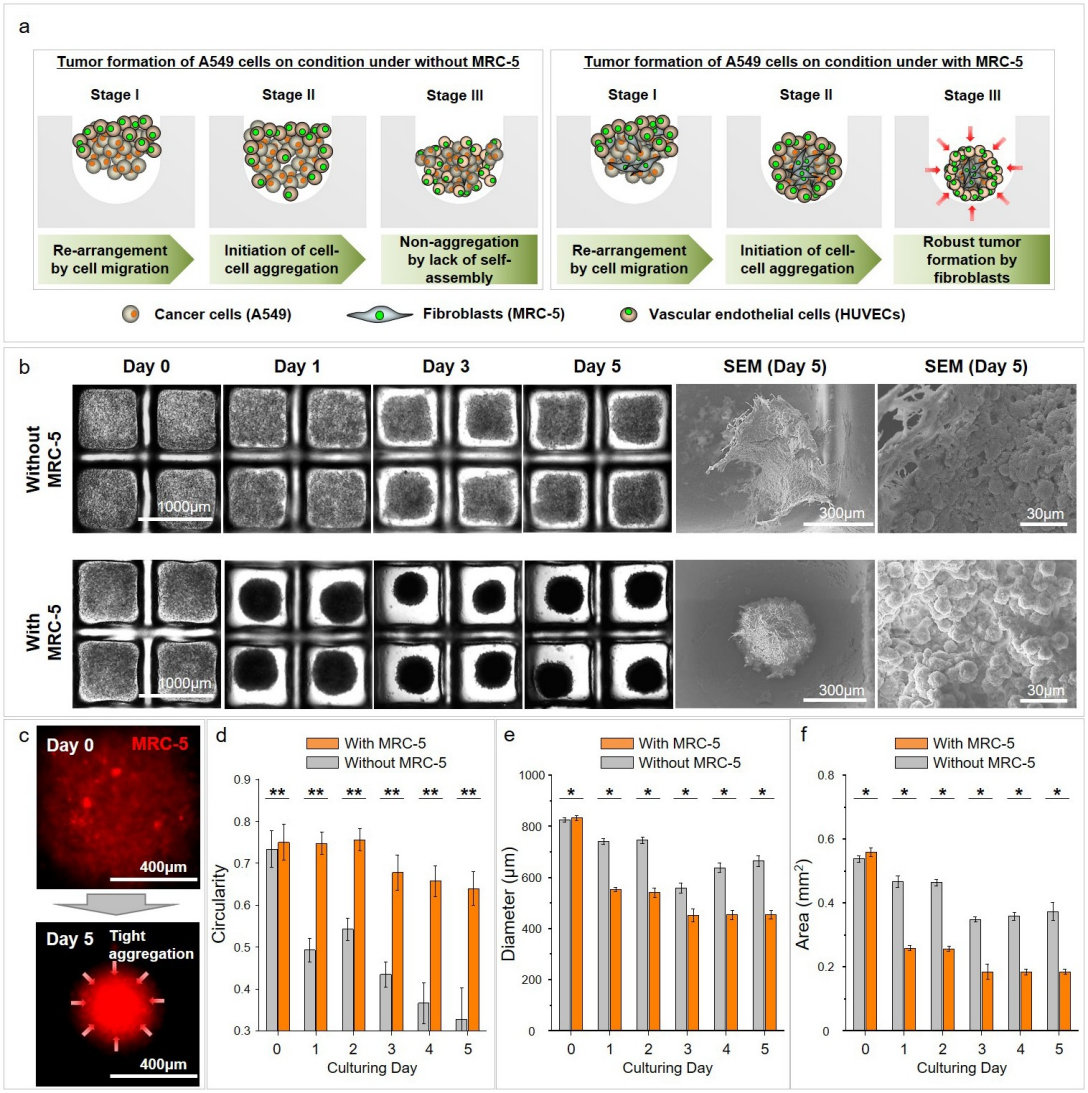
Process and morphological change for robust MCTs formation of cancer cells by fibroblasts on CLF concave microwell array. (a) MCT formation processes without and with the MRC–5 groups on the CLF concave microwell array. (b) Tumoroid formation during cultivation on the CLF concave microwell array, and SEM image used for the identification of cell-to-cell interactions. (c) MRC–5 cell localization using a cell tracker in the cell mixture during tumoroids formation. The circularity (d), diameter (e) and area (f) of MCTs on culturing days. *p* < 0.05(*) and *p* < 0.005(**).

We produced a large number of MCTs and investigated their formation and features. A mixture of A549, MRC-5 and HUVECs cells was seeded onto the CLF concave microwell arrays without any other processes (S2(a) and S2(b) Fig). The cell mixture rapidly and spontaneously aggregated to spherical form within one day. Fig 2(a) demonstrates the process of MCTs formation in a group with fibroblasts. During the MCTs formation, partial aggregation is observed in the presence of only A549 cancer cells as a comparative group, but robust MCTs formation occurs through the three-step processes for the A549, MRC-5 and HUVECs mixture group as an experimental group: i) re-arrangement by cell migration, ii) initiation of aggregation by fibroblasts, and iii) robust tumor formation by fibroblasts (Fig 2(a)). The initial aggregation and MCTs formation occurred within one day, and robust type was formed from days 3 in the CLF concave microwell array compared with MCTs without MRC-5 cells. The SEM image shows strong cell-to-cell interactions within the MRC-5 group (Fig 2(b)). In particular, cell localization was confirmed by the cell tracker in the cell mixture, and MRC–5 fibroblasts gradually localized to the central positions of the MCTs during culturing and induced tight aggregation of the cell mixture (Fig 2(c) and S5 Fig). The circularity of the MCTs was evaluated, and the morphology of MCTs with MRC-5 cells were spherical shape (Fig 2(d)). In addition to this circularity result, the diameter and area of the MCTs group with MRC-5 was markedly decreased from day 1 and gradually decreased more than the without MRC-5 group until day 5 of culture (Fig 2(e) and 2(f)). The size of the MCTs is approximately half the size of the microwell diameter. Therefore, these robust MCTs formation can be considered to be due to the influence of MRC-5 fibroblasts. The CLF concave microwell is a useful tool for human lung MCTs formation.

### Comparison of αSMA as a myofibroblast marker through TGFβ activation by fibroblasts in MCTs

In the TME, TGFβ is known as a key factor that induces trans-differentiation of cancer resident fibroblasts into αSMA-positive myofibroblasts, such as cancer-associated fibroblasts (CAFs), and promotes tumor progression. Therefore, we confirmed expression of transforming growth factor-beta (TGFβ) and alpha smooth muscle actin (αSMA) to identify activation of tumorigenesis by the trans-differentiation of cancer resident fibroblasts into myofibroblasts.

**Fig 3:**
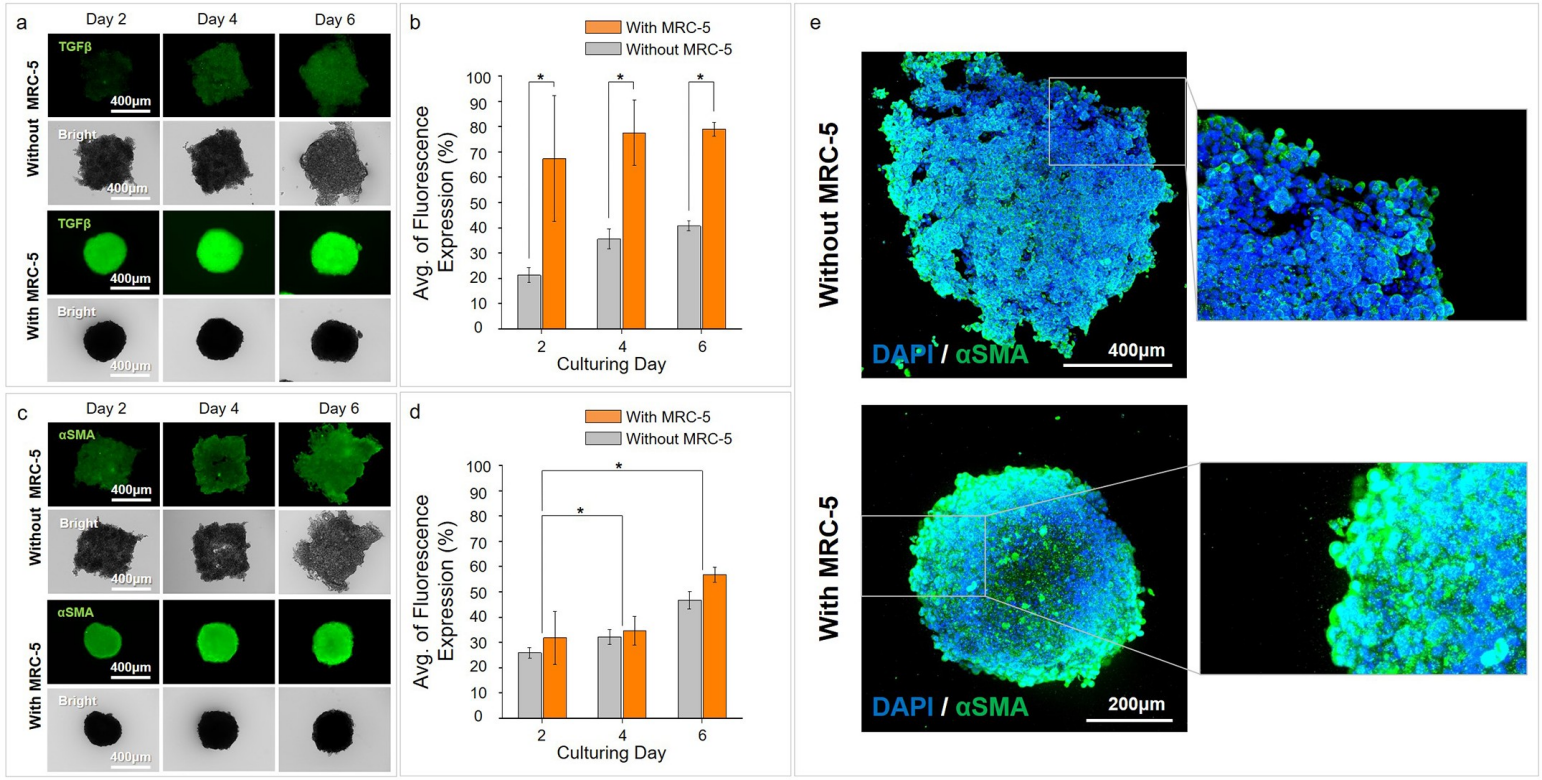
High expression of αSMA, a myofibroblast marker, through TGFβ activation by fibroblasts in MCTs. (a, b) TGFβ expressions without and with the MRC–5 groups on the CLF concave microwell array. (c, d) αSMA expression without and with the MRC–5 groups on the CLF concave microwell array. (e) Confocal images confirm the expression of αSMA. *p* < 0.05(*).

Expression of TGFβ in the without MRC-5 group showed weak expression in the partial aggregation area for all culture days. However, in the condition with MRC-5, TGFβ was highly expressed from an early culture day and was persistently expressed during the culture days (Fig 3(a) and 3(b)). In particular, TGFβ expression was detected in most areas of the tri-cultured MCTs from day 6, although it was weakly positive within the MCTs at day 2 (Fig 3(a)).

The expression pattern of αSMA appeared differently than the expression pattern of TGFβ. When MCTs with and without MRC-5 cells were compared the increase in αSMA in the MRC-5 group was not as large as the increase in TGFβ. In the without MRC-5 group, the expression of αSMA was also observed in the partial aggregation area. In addition, the expression increased with time. Similarly, in the MRC-5 group, the expression of αSMA was higher than that without MRC–5 but with a similar pattern. Moreover, a time-dependent increase was also confirmed (Fig 3(c), 3(d) and 3(e)). The time dependent increases in αSMA expression have significant differences. These results of αSMA by TGFβ expression under the conditions with and without MRC-5 cells suggest that MRC-5 fibroblasts were trans-differentiated into myofibroblasts by the secretion of TGFβ from A549 cancer cells.

### Comparison of ECM related markers in MCTs

**Fig 4:**
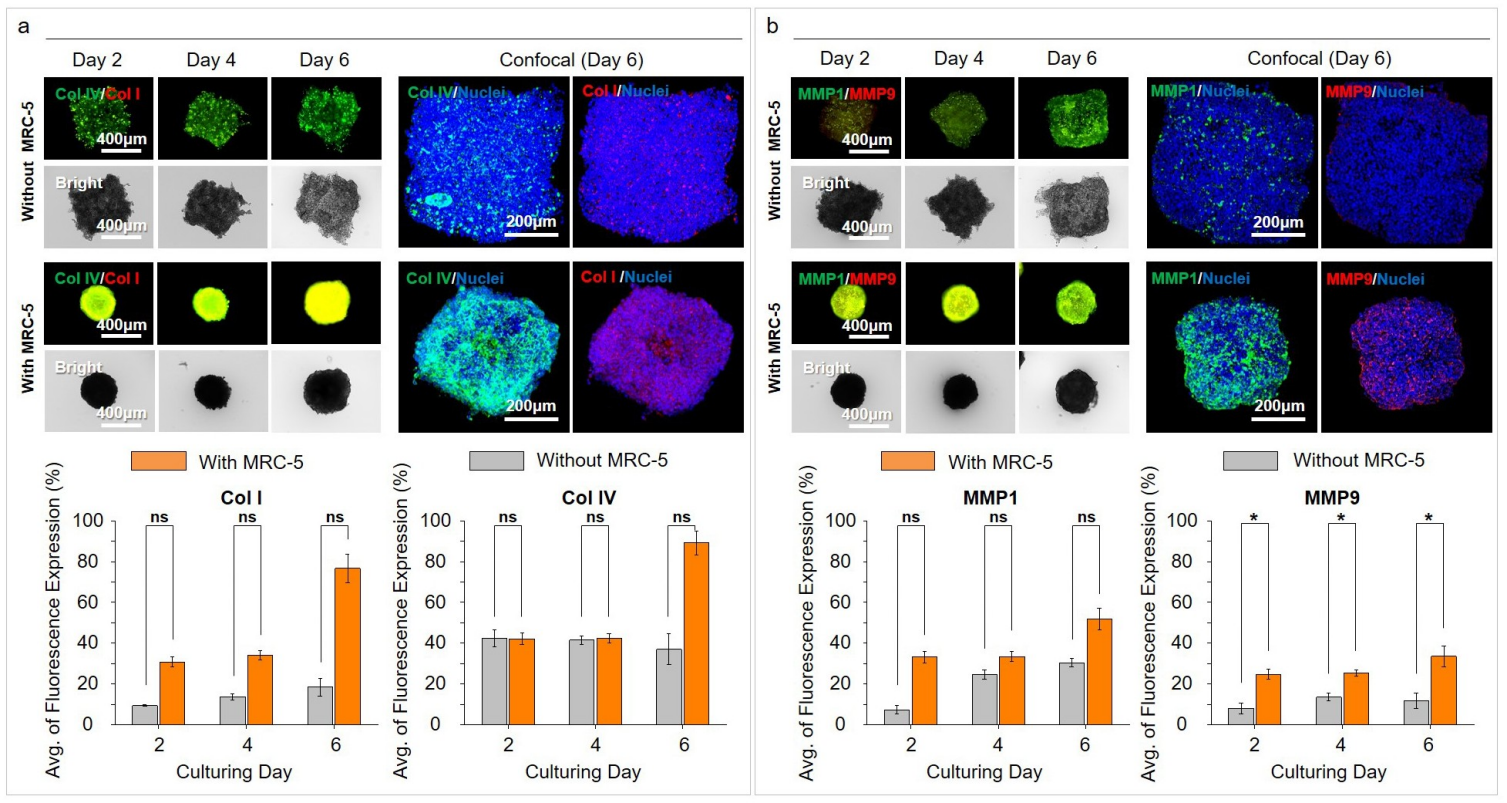
Expression of ECM related markers in MCTs. (a) Expression of collagen types I and IV without and with the MRC–5 groups in the CLF concave microwell array. (b) Expression of MMP1 and MMP9 without and with the MRC–5 groups on the CLF concave microwell array. *p* > 0.05(ns), *p* < 0.05(*).

To identify the matrix formation of the MCTs, we conducted collagen type I and IV immunostaining of the MCTs with and without MRC-5 cells. Expression of collagen type I and IV as ECM components during the formation of the MCTs was significantly different between the without and with MRC-5 cells groups (Fig 4(a)). In the MCTs without MRC-5 cells, collagen type I expression showed initially weak positive, and it showed a weak increase in the overall area on day 6. Collagen type IV expression was positive in the high cell aggregation area during tumorigenesis, and increased with the augmentation of the aggregation area. In the group with MRC-5 cells, collagen type IV showed high expression in the outer layer of MCTs aggregating cancer cells during rapid MCTs formation. It appeared very high expression in the overall space of robust MCTs on day 6. In this group, collagen type I was expressed in most areas of the MCTs and highly increased on day 6. Confocal images showed the detailed level of these collagen expression. Type I and IV collagen was activated in the whole area of the MCTs model with MRC-5.

In addition, we assessed the expression of MMP1 and MMP9 to identify ECM enzymes during tumorigenesis in the MCTs (Fig 4(b)). Expression of MMP1 and MMP9 was also significantly different between the two groups. In MCTs without MRC-5, MMP1 and 9 expressions showed weak positive on early time and appeared a weak increase depend on cultivation period. However, in MCTs with MRC-5 cells, MMP1 showed a stagnated expression pattern with weak expression until day 4, but increased expression on day 6. MMP9 expression was also observed a similar pattern with that of MMP1 until day 6. Expression of collagen and MMPs exhibited a similar increase in patterns.

### Comparison of angiogenesis-related markers in the MCTs

**Fig 5:**
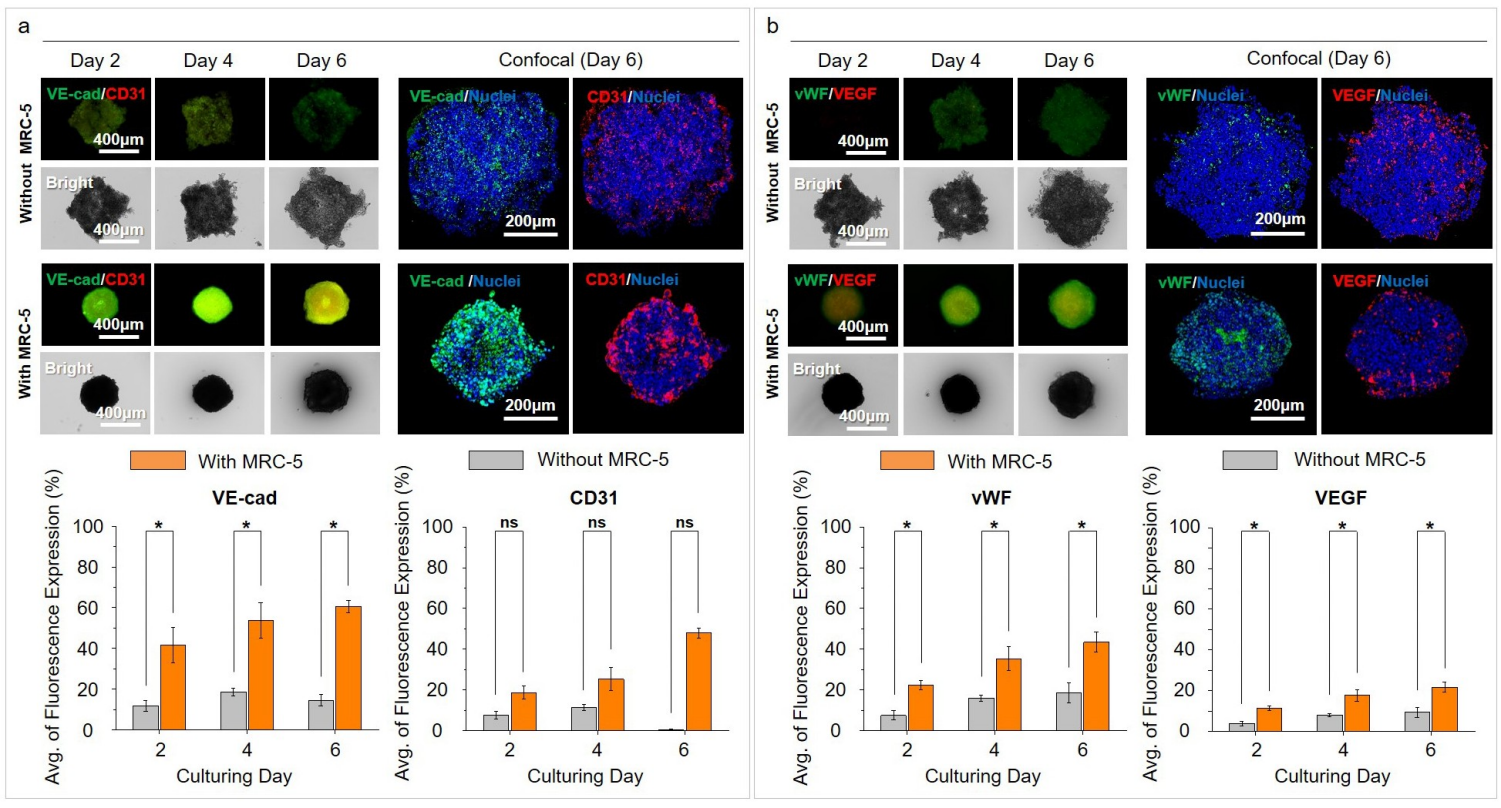
Expression of angiogenesis related markers in MCTs. (a) Expressions of VE-cad and CD31 without and with the MRC–5 groups in the CLF concave microwell array. (b) Expressions of vWF and VEGF without and with the MRC–5 groups on CLF concave microwell array. *p* > 0.05(ns), *p* < 0.05(*).

We assessed the expression of markers related to angiogenesis for the possibility of the development of tumor vasculature. VE-cadherin (VE-cad) and CD31 showed a difference in expression levels and patterns between MCTs without and with MRC-5 cells. In MCTs without MRC-5 cells, the increased expression of VE-cad and CD31 were continuously identified until day 4, but decreased on day 6. VE-cad and CD31 expression in MCTs with MRC-5 was continuously identified with a time dependent increase until day 6 (Fig 5(a)). von Willebrand factor (vWF) and vascular endothelial growth factor (VEGF) were weakly expressed in MCTs without MRC-5 cells. In MCTs with MRC-5 cells, vWF and VEGF expression continuously increased until day 6. These results suggest that MCTs develop more rapidly with rapid aggregation in the presence of MRC-5 cells (Fig 5(b)).

### Drug response to anticancer agents in MCTs

To identify the anti-cancer drug response of MCTs robustly formed by cancer resident fibroblasts (MRC-5) in fabricated CLF concave microwells, various agents that target lung cancer were administered for 4 days. To determine the dose of the anticancer drug, we measured the IC50 using a CCK–9 cytotoxicity assay kit after treatment with Paclitaxel and Gemcitabine at various doses (0.39, 1.56, 6.25, 25, and 100 μM) for a period of four days (S6 Fig). As a result, Gemcitabine exhibited a slight error value on the data of day 3 after the drug administration but yielded IC50 values of ~3 μM after four days (day 1, 2.4 μM; day 2, 9.2 μM; day 3, 2.93 μM; day 4, 3.05 μM). IC50 values of Paclitaxel were significantly higher than Gemcitabine and decreased gradually over a period of four days (day 1, 35.8 μM; day 2, 25.6 μM; day 3, 22.6 μM; day 4, 22.8 μM). Based on these results, we tried to confirm the synergistic effects of additional drugs by applying the IC50 values of Gemcitabine to Paclitaxel.

**Fig 6:**
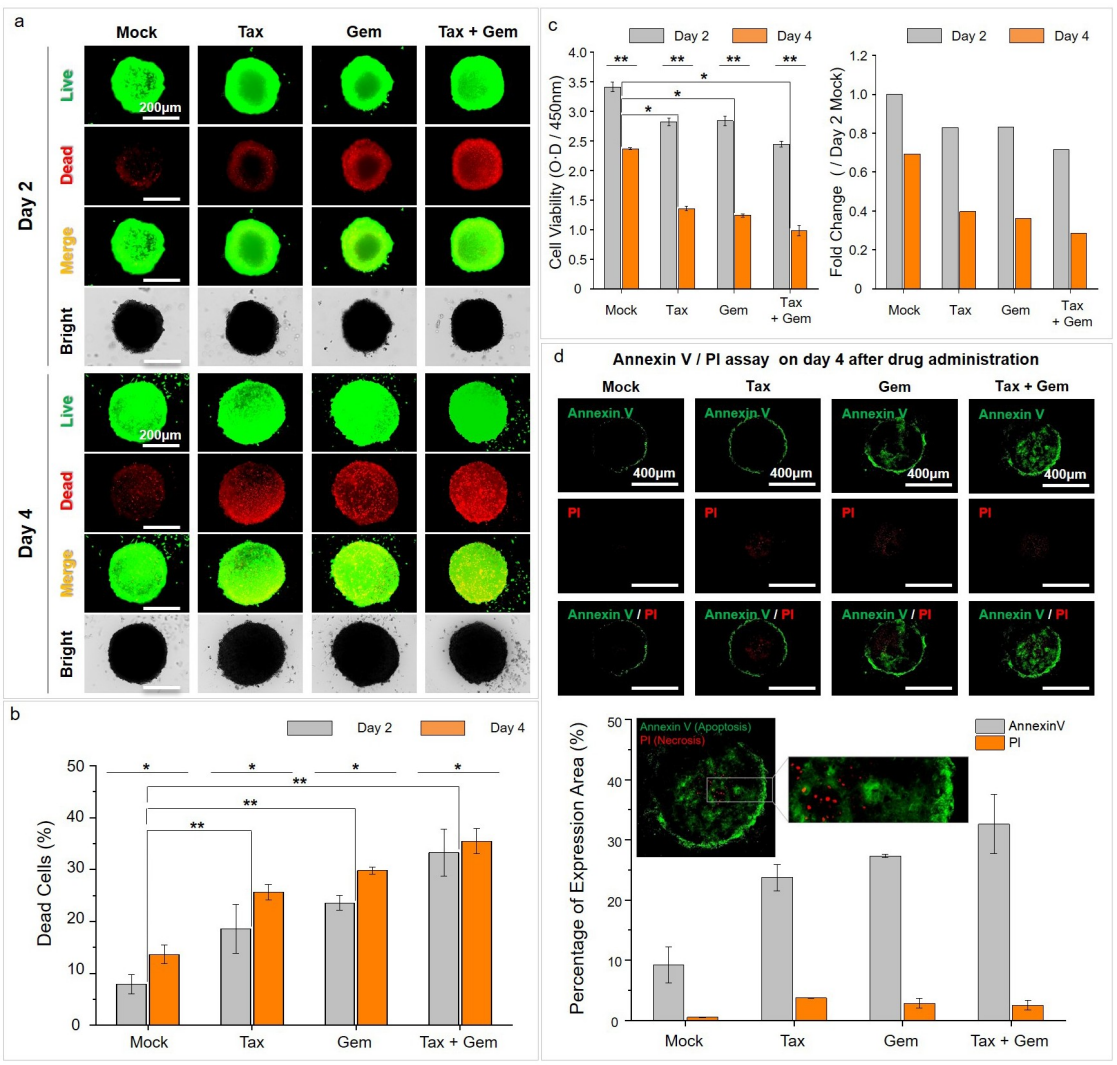
Drug response to anticancer agents in MCTs with MRC-5. (a) Staining assay of live and dead cells with use of calcein AM (live cells) and ethidium bromide (dead cells) (b) Percentages of dead cells based on the measurements of the fluorescence intensities of live/dead cells using ImageJ on day 2 and 4 after the initiation of drug administration. (c) Cell viability was performed using the CCK–8 assay kit and was measured at 450 nm using a microplate reader on day 2 and 4 after the initiation of drug administration. (d) Annexin V and PI staining were used to identify the apoptotic cell death after drug administration. *p* < 0.05(*) and *p* < 0.005(**).

The staining assay was used to confirm the ALDH activity on the fourth day after the onset of the drug administration. As shown, ALDH was very weakly expressed in all the groups administered with anti-cancer drugs. This demonstrates that the response to the drug treatment can be effective up to the central region of the 3D MCTs in all the groups (S7 Fig). Fig 6(a) was captured following the fluorescent labeling of both live and dead cells with calcein AM and ethidium bromide, respectively. Administration of all anti-cancer agents induced cell death from day 2. Moreover, administration of the combination (Taxol (paclitaxel) +Gemcitabine) led to higher cell death than Taxol (paclitaxel) or gemcitabine single: Taxol (paclitaxel) (<20%), Gemcitabine (<25%), Taxol (paclitaxel) + Gemcitabine (<35%). On day 4, the administration of all anti-cancer agents induced the time dependent cell death. However, in the untreated group, dead cells also increased in a time-dependent manner (Fig 6(b)). The percentage of dead cells was performed to verify the pattern of dead cells by fluorescence intensity. In this analysis, similar to the increase of dead cells in fluorescence intensity, cell viability decreased with the administration of anti-cancer drugs. In addition, cell viability in the mock, untreated group also had a time-dependent decrease (Fig 6(c)). Consideration of the results that confirm the apoptotic cell death induced by the drug response on day 4 after the onset of the drug administration shows that although the necrotic area was weakly detected in the center region of the MCTs, the highest proportion of the cell death area was attributed to the apoptotic death due to the drug response (Fig 6(d)). This apoptotic pattern was then compared with the percentage of the areas occupied by dead cells. Although there was a slight difference in the mock comparing with anti-cancer drug administration groups, it was confirmed that a similar dead cell percentage pattern can be obtained by minimizing cell loss in repeated experiments using CLF microwell arrays. Based on the comparisons, it is determined that the experimental reproducibility is adequate given the similarities of the cell loss minimization data (S8 Fig).

## Discussion

The formation of 3D spheroids using micro-techniques has been carried out by many researchers to create the *in vitro* tumor models similar to the *in vivo* tissue for an array for cancer research [17, 37, 38]. This method for producing a large number of 3D MCTs using multi-cell lines and patient-derived cells is a promising technology for effective drug application and discovery of new drugs for therapeutic purposes [17, 39]. For this reason, the development of a suitable structure, capable of the formation of numerous, similar 3D MCTs under the same conditions, has great advantages for these studies [40, 41]. Furthermore, spherical shape and homogeneous size of the MCTs can affect the drug cytotoxicity test [38]. They tested the assay on different-sized spheroids (diameter: 350 ~ 850 μm), then obtained a stable linear increment in luminescence by reagent penetration as spheroid size increased from 350 to 600 μm. Although, the conventional microtechnology-based microstructures are commonly used for the formation of homogeneous size and meaningful level of *in vitro* 3D tumors [42, 43], microwell with a spiky wall has advantages during the process of the cell seeding for cell spheroid formation (S1 and S2 Figs, S1 Movie). These spiky walls were formed by a simple process during the fabrication of the CLF microwell array, and allowed cells to flow through the microwell during the initial seeding process depending on gravity. The pattern-carved PMMA sheet can fabricate size-controlled and high-density microwell arrays (697 wells per plate (well diameter = 1.0 mm) within ø35 mm plate). Using the CLF well arrays, we represent it was possible to obtain spherical and uniform MCTs of approximately 450 μm size in 3 days. We demonstrate the CLF microwell arrays are suitable to fabricate the large MCTs with homogeneous size which contribute to the reduction of data variability for drug testing through the formation of numerous similar sized MCTs with minimizing cell loss.

As TME resident fibroblasts, CAF regulates the biophysical and biochemical properties of the TME. CAFs can promote the initiation and development of tumor formation [44, 45]. The used MRC-5 cell line was reported that can play a role of CAFs in studies [46–48]. We aimed to represent the *in vitro* custom-shaped heterotypic tumoroid model within the CLF microwell array. The mixture of cancer cells (A549), vascular endothelial cells (HUVECs), and fibroblasts (MRC-5) were plated on the fabricated CLF concave microwells to develop the *in vitro* MCTs model. The spherical MCTs spontaneously formed within 1 day in the CLF microwells. During the MCTs formation, MRC-5 was highly responsible for tumorigenesis including spherical morphology and formation time. Identification of drug response along with the formation of 3D MCTs is an important part of the purpose of an *in vitro* tumor model [37, 38]. To evaluate the drug response of the 3D tumor model, Taxol (paclitaxel) and gemcitabine (anti-cancer agents for non-small cell lung cancer) were directly applied to the MCTs formed under the condition with MRC-5 cells in CLF concave microwell array. Here, the presence of fibroblasts led to the formation of a compact matrix, and the robust formation of the MCTs.

## Conclusions

We have established a CLF concave microwell arrays with using laser carving for the production of molds to form numerous human lung MCTs capable of drug screening. By utilizing the CLF concave microwell array fabricated by this technology, the biophysicochemical effect of TME resident fibroblasts was demonstrated, and the production of MCTs was similar to that of the 3D *in vitro* tumor models created by the conventional method. The formation of large numbers of MCTs with meaningful size and spherical morphology also yielded significant results in confirming drug response for anti-cancer drug screening.

## Supporting information

S1 Fig Fabrication of PDMS based CLF microwell array by laser carved PMMA master mold. Procedure for fabrication of the PDMS cell-loss-free (CLF) microwell array by pattern carving utilizing a laser cutter.
(TIF)

S2 Fig Procedure of cell application for MCTs in CLF concave microwell array. (a) The pointed oval-shaped edge allows slippage of plated cells along the partition wall by gravity. Thus, the CLF microwell array does not require wash out or any other processes. (b) To form MCTs using the CLF concave microwell arrays, A549 lung carcinoma cells, HUVECs, and MRC-5 cells which served as cancer resident fibroblasts, were pre-mixed and plated on the fabricated CLF concave microwell array. The cell mixture was settled into the bottom of the wells using a centrifuge. After settlement of the cell mixture, it was incubated.
(TIF)

S3 Fig Size control on the fabrication of cell-loss-free (CLF) concave microwell array. SEM images identified that the control of the size and structural design can be adjusted.
(TIF)

S4 Fig Stable cell trapping in CLF concave microwell array by minimizing cell loss. (a) Stable cell trapping by spiky walls in CLF microwell array. (b) Observation for confirmation of stable cell trapping using tracker labeled cells. (c) Exchange of cell area after initial cell trapping.
(TIF)

S5 Fig Central localization of MRC-5 cells during the process of MCTs formation in CLF concave microwell array. Cell localization was confirmed using cell tracker in the cell mixture in CLF concave microwell array during MCTs formation, and MRC-5 fibroblasts gradually localized to the central position of the MCTs during the time period and induced tight aggregation of the cell mixture.
(TIF)

S6 Fig IC50 test to determine dose of anti-cancer drugs. IC50 assay after treatments with Paclitaxel and Gemcitabine at various doses (0.39, 1.56, 6.25, 25, and 100 μM) for four days.
(TIF)

S7 Fig Staining assay used to confirm the ALDH activity. ALDH activity on day 4 after the onset of anticancer drug administration.
(TIF)

S8 Fig Comparative analysis for the verification of reproducibility. Comparison of dead cell and apoptotic/necrotic cell areas on day 4 after the drug administration.
(TIF)

S1 Movie Minimization of cell loss due to sliding of cells by spiky walls in CLF microwell array. Observations for 20 minutes after cell trapping.
(MP4)
