# Supplementary figures and images for: A cell-loss-free concave microwell array based size-controlled multi-cellular tumoroid generation for anti-cancer drug screening
Source: PLoS One. 2019 Jul 25;14(7):e0219834. doi: 10.1371/journal.pone.0219834 (PMC6658056; doi:10.1371/journal.pone.0219834)

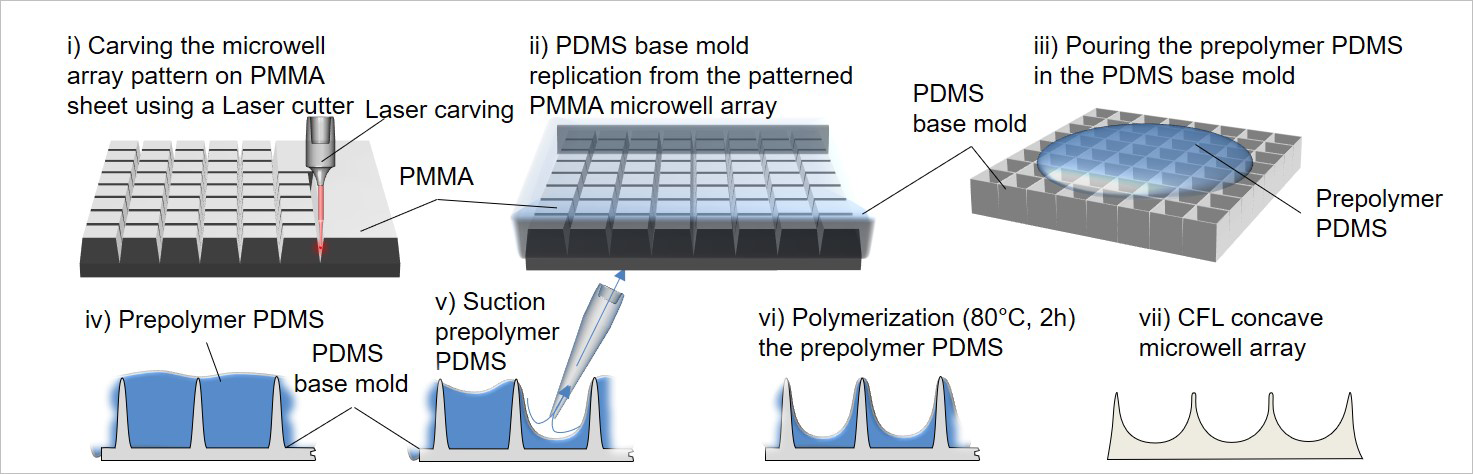

Supplement: S1 Fig — Procedure for fabrication of the PDMS cell-loss-free (CLF) microwell array by pattern carving utilizing a laser cutter. (TIF) [file pone.0219834.s001.tif]

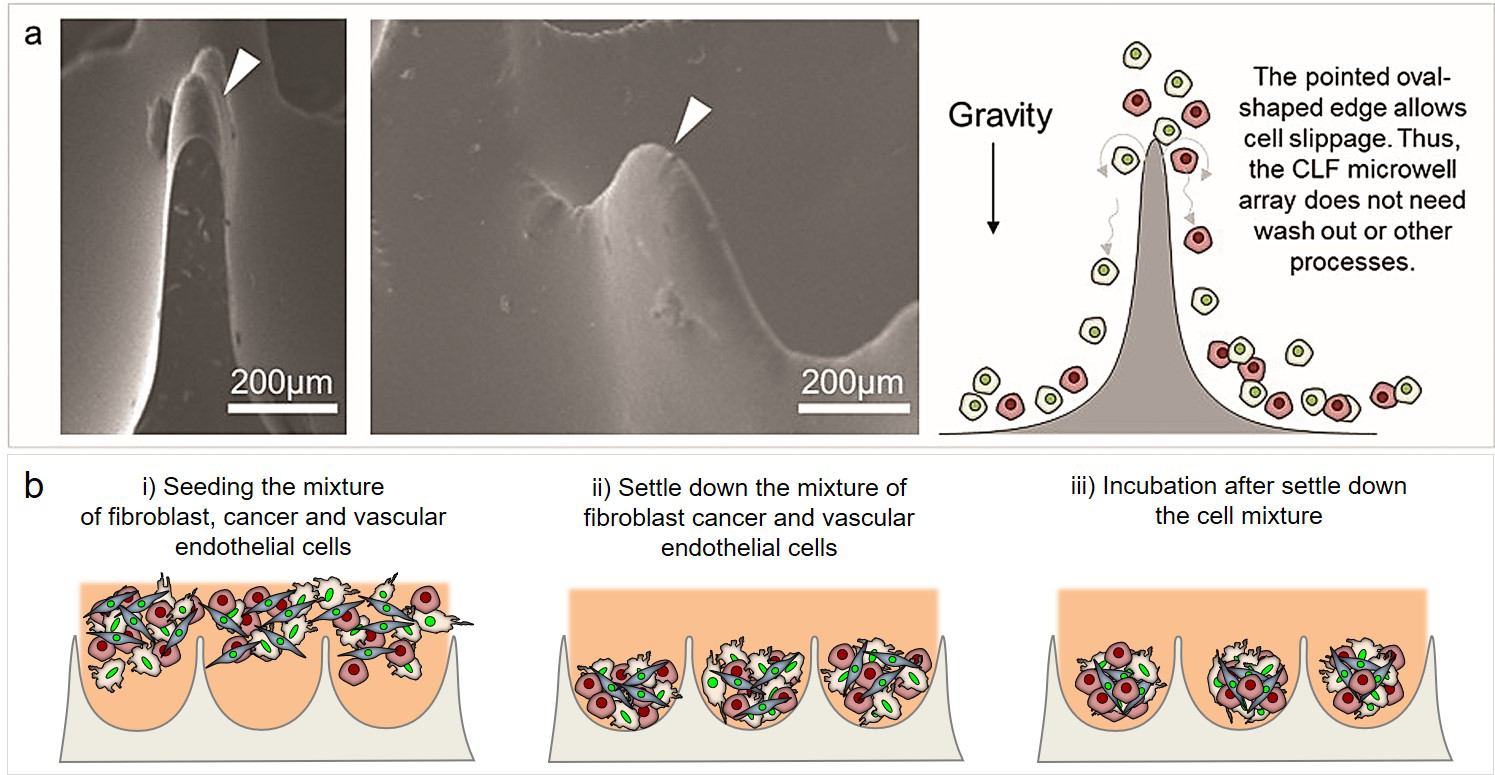

Supplement: S2 Fig — (a) The pointed oval-shaped edge allows slippage of plated cells along the partition wall by gravity. Thus, the CLF microwell array does not require wash out or any other processes. (b) To form MCTs using the CLF concave microwell arrays, A549 lung carcinoma cells, HUVECs, and MRC-5 cells which served as cancer resident fibroblasts, were pre-mixed and plated on the fabricated CLF concave microwell array. The cell mixture was settled into the bottom of the wells using a centrifuge. After settlement of the cell mixture, it was incubated. (TIF) [file pone.0219834.s002.tif]

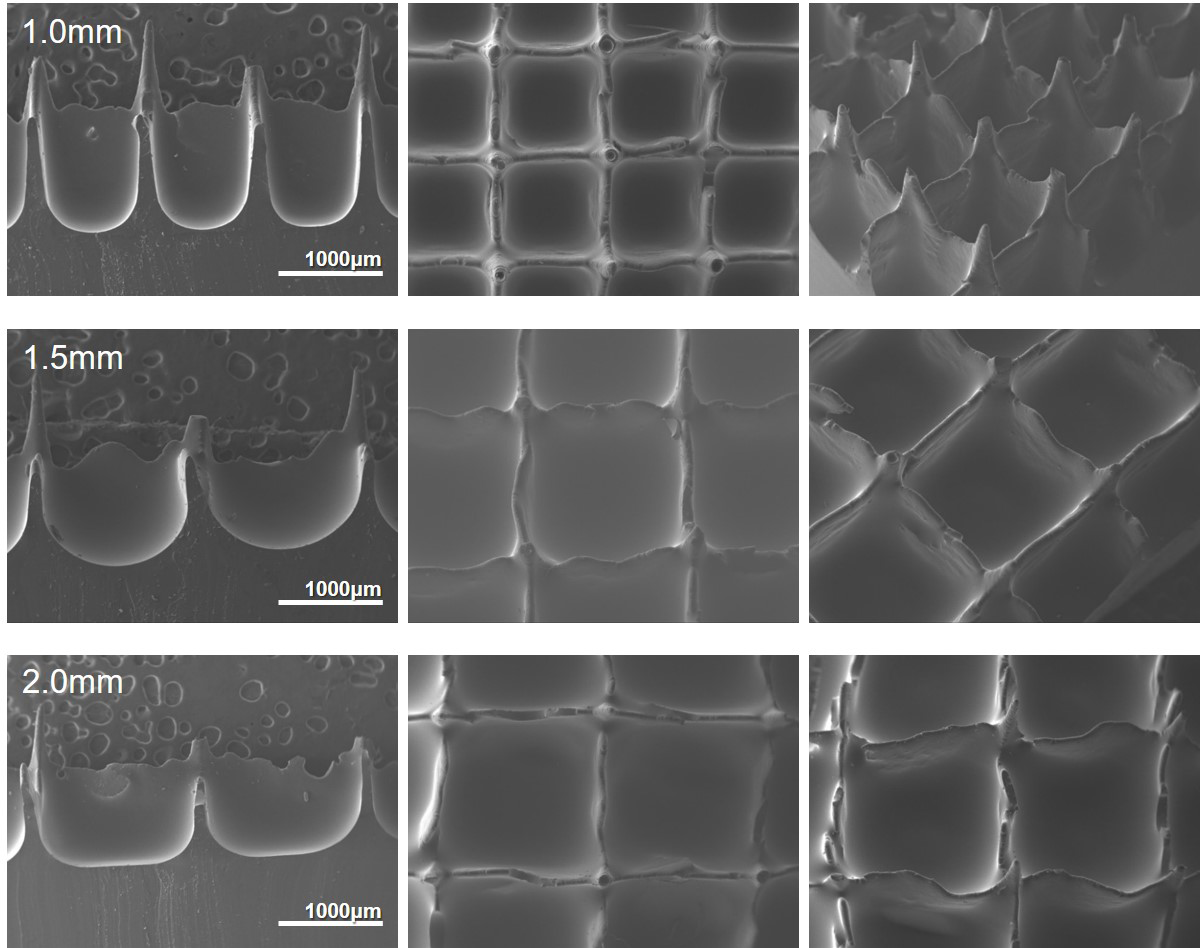

Supplement: S3 Fig — SEM images identified that the control of the size and structural design can be adjusted. (TIF) [file pone.0219834.s003.tif]

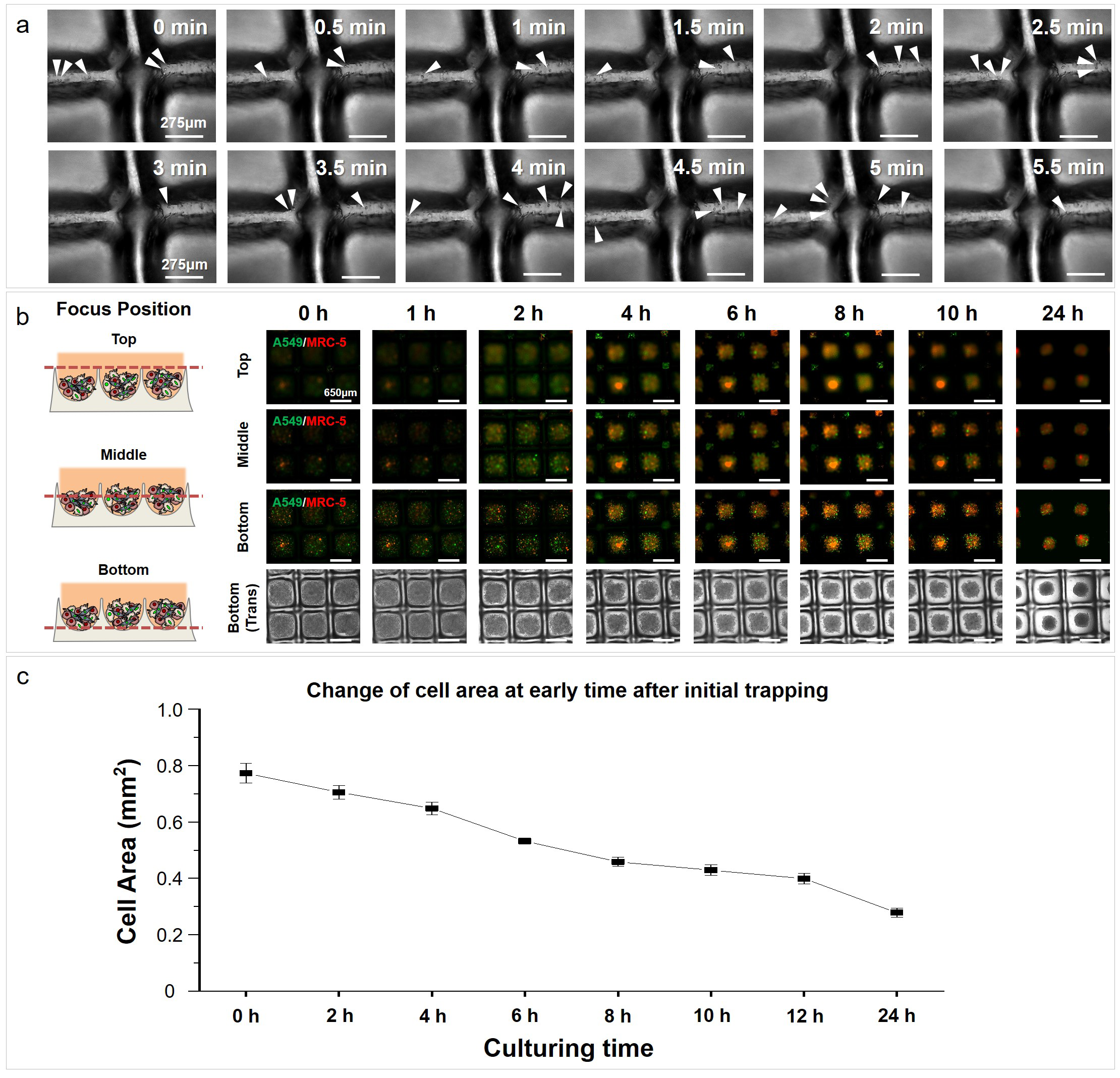

Supplement: S4 Fig — (a) Stable cell trapping by spiky walls in CLF microwell array. (b) Observation for confirmation of stable cell trapping using tracker labeled cells. (c) Exchange of cell area after initial cell trapping. (TIF) [file pone.0219834.s004.tif]

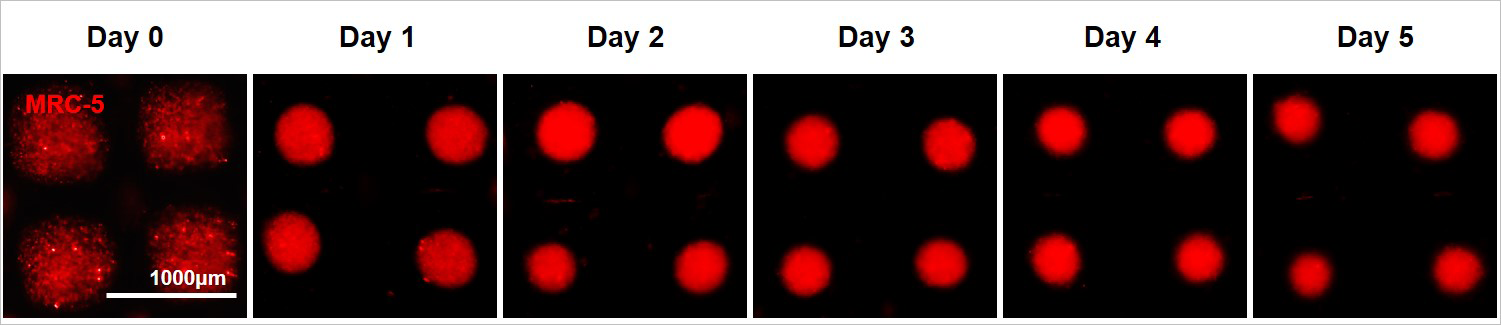

Supplement: S5 Fig — Cell localization was confirmed using cell tracker in the cell mixture in CLF concave microwell array during MCTs formation, and MRC-5 fibroblasts gradually localized to the central position of the MCTs during the time period and induced tight aggregation of the cell mixture. (TIF) [file pone.0219834.s005.tif]

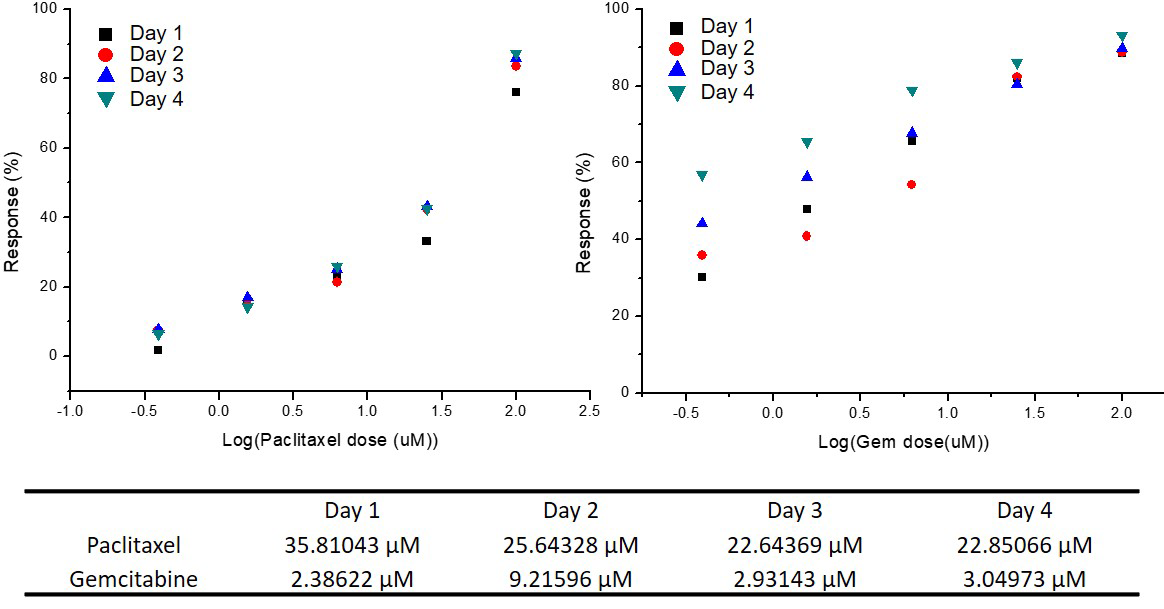

Supplement: S6 Fig — IC50 assay after treatments with Paclitaxel and Gemcitabine at various doses (0.39, 1.56, 6.25, 25, and 100 μM) for four days. (TIF) [file pone.0219834.s006.tif]

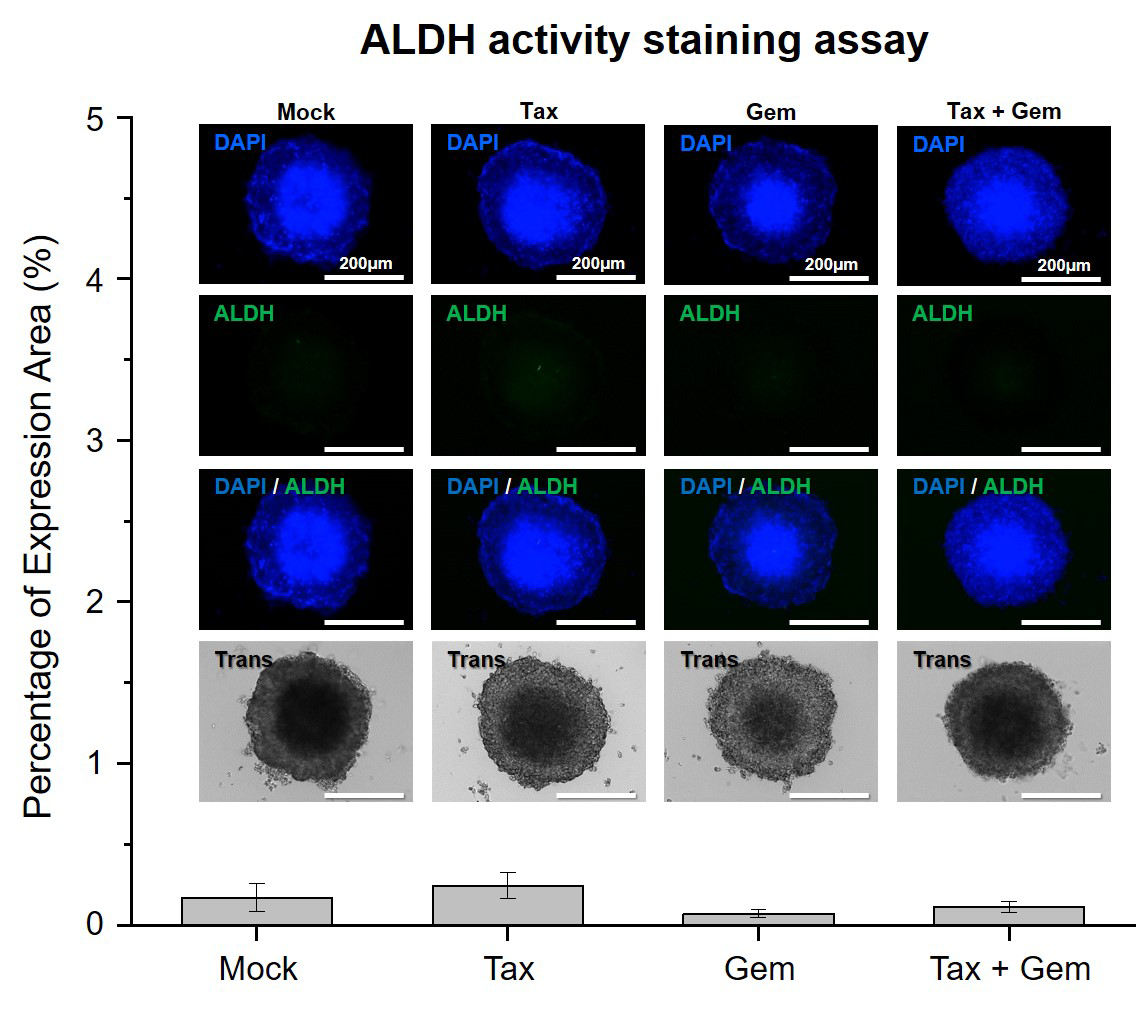

Supplement: S7 Fig — ALDH activity on day 4 after the onset of anticancer drug administration. (TIF) [file pone.0219834.s007.tif]

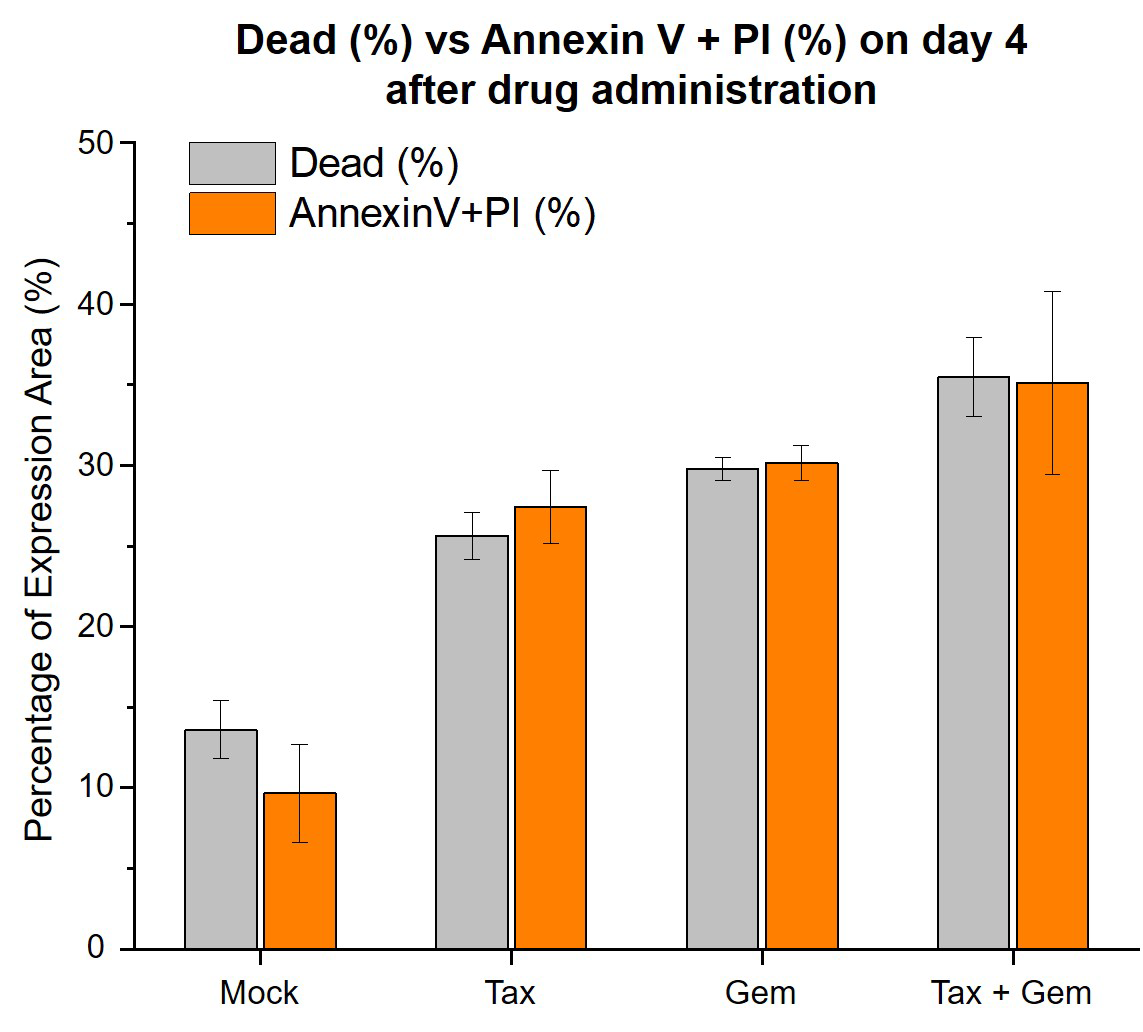

Supplement: S8 Fig — Comparison of dead cell and apoptotic/necrotic cell areas on day 4 after the drug administration. (TIF) [file pone.0219834.s008.tif]
